# Attributable Causes of Esophageal Cancer Incidence and Mortality in China

**DOI:** 10.1371/journal.pone.0042281

**Published:** 2012-08-02

**Authors:** Jian-Bing Wang, Jin-Hu Fan, Hao Liang, Jing Li, Hui-Juan Xiao, Wen-Qiang Wei, Sanford M. Dawsey, You-Lin Qiao, Paolo Boffetta

**Affiliations:** 1 Department of Cancer Epidemiology, Cancer Institute/Hospital, Chinese Academy of Medical Sciences, Peking Union Medical College, Beijing, China; 2 Division of Cancer Epidemiology and Genetics, Nutritional Epidemiology Branch, National Cancer Institute, Bethesda, Maryland, United States of America; 3 Nutrition Department, Tianjin Third Central Hospital, Tianjin, China; 4 The Tisch Cancer Institute, Mount Sinai School of Medicine, New York, New York, United States of America; 5 International Prevention Research Institute, Lafayette, Lyon, France; Baylor College of Medicine, United States of America

## Abstract

**Background:**

To estimate the contribution of tobacco smoking, alcohol drinking, low vegetable intake and low fruit intake to esophageal cancer mortality and incidence in China.

**Methodology/Principal Findings:**

We calculated the proportion of esophageal cancer attributable to four known modifiable risk factors [population attributable fraction (PAF)]. Exposure data was taken from meta-analyses and large-scale national surveys of representative samples of the Chinese population. Data on relative risks were also from meta-analyses and large-scale prospective studies. Esophageal cancer mortality and incidence came from the 3^rd^ national death cause survey and population-based cancer registries in China. We estimated that 87,065 esophageal cancer deaths (men 67,686; women: 19,379) and 108,206 cases (men: 83,968, women: 24,238) were attributable to tobacco smoking, alcohol drinking, low vegetable intake and low fruit intake in China in 2005. About 17.9% of esophageal cancer deaths among men and 1.9% among women were attributable to tobacco smoking. About 15.2% of esophageal cancer deaths in men and 1.3% in women were caused by alcohol drinking. Low vegetable intake was responsible for 4.3% esophageal cancer deaths in men and 4.1% in women. The fraction of esophageal cancer deaths attributable to low fruit intake was 27.1% in men and 28.0% in women. Overall, 46% of esophageal cancers (51% in men and 33% in women) were attributable to these four modifiable risk factors.

**Conclusions/Significance:**

Tobacco smoking, alcohol drinking, low vegetable intake and low fruit intake were responsible for 46% of esophageal cancer mortality and incidence in China in 2005. These findings provide useful data for developing guidelines for esophageal cancer prevention and control in China.

## Introduction

Esophageal cancer is the 8^th^ most common cancer worldwide, with 482,000 new cases in 2008, and the 6^th^ most common cause of death from cancer, with 406,000 deaths (5.4% of all cancer deaths) [1]. Over 80% of esophageal cancers occur in developing countries, where nearly all cases are esophageal squamous cell carcinoma (ESCC). In China, the crude mortality rate of esophageal cancer in 2004–2005 was 15.2/100,000, which represented 11.2% of all cancer deaths and ranked as the 4^th^ most common cause of cancer death [2]. Thus, even though the age-standardized mortality of esophageal cancer decreased by 41.6% from 1973 to 2005 [3], China still suffers a great disease burden from esophageal cancer.

Over the past few decades, a number of risk factors for ESCC have been identified, including tobacco smoking, alcohol drinking, dietary and micronutrient deficiency, high temperature of beverage and food consumption, poverty, history of head and neck cancer and other miscellaneous factors (such as fast eating habits and polycyclic aromatic hydrocarbons (PAH) exposure) [4]. Tobacco smoking and alcohol drinking are major risk factors for ESCC in the US population, explaining over 90% of cases in men [5]. But several epidemiological studies have shown that smoking and alcohol drinking play a much less significant role in the etiology of esophageal cancer in the high risk areas of China [6], [7].

Several studies have reported the attributable causes of cancer in western populations [8], [9], [10]. For example, the burden of cancer mortality and incidence attributable to risk factors was estimated in France in 2000 [8], and it was found that the fraction of esophageal cancer deaths caused by tobacco smoking, alcohol drinking, overweight and obesity was 79.2% for men and 49.4% for women. Only one case control study, focusing on one province, has estimated the proportion of esophageal cancer attributable to lifestyle risk factors in China, and it found the combined population attributable fraction (PAF) to be 62.6% [11].

To estimate attributable causes of esophageal cancer in China is crucially important for esophageal cancer control and prevention. The objective of our study was to provide an evidence-based assessment of the proportion of esophageal cancer cases and deaths attributable to tobacco smoking, alcohol drinking, and low vegetable and fruit intake in China in 2005.

## Methods

### Overview

Our study was one part of a project called “attributable causes of cancer in China”, which aims to estimate the contribution of known causes of cancer, including smoking [12], alcohol drinking [13], chronic infection [14], nutritional factors [15], overweight and obesity, occupational factors and hormonal factors on the cancer burden in China. This report provides an evidence-based assessment of numbers and proportions [population attributable fractions (PAFs)] of esophageal cancer deaths and cases in China in 2005 that could be attributable to tobacco smoking, alcohol drinking, low vegetable intake and low fruit intake. Since part of the material (PAF for smoking [12], alcohol drinking [13] and nutritional factors [15]) has already been published, this paper focuses on the joint effects of these risk factors on esophageal cancer and a comparison of these joint effects with those found in other similar studies.

PAF was defined as the amount of the cancer burden in a population which could be eliminated by modifying or removing the exposure of certain causal factors. In our study, the estimated PAF was calculated based on the counterfactual scenario of total avoidance of risk factors (such as smoking and alcohol drinking). For some other risk factors (low vegetable and fruit intake), zero exposure was inappropriate, so we estimated the fraction of esophageal cancer that would not have occurred under an alternate scenario of level, frequency or intensity of exposure.

Our estimates were restricted to esophageal squamous cell carcinoma because esophageal adenocarcinoma is rare in China and few data are available at the national level.

### Cancer Mortality and Incidence Data

Cancer mortality data were derived from the 3^rd^ National Death Cause Survey in China [2]. Briefly, this was a retrospective survey conducted in 160 randomized counties and 53 cancer high-risk areas between 2004 and 2005.

Cancer incidence data were estimated by using a Mortality/Incidence (M/I) ratio and the known cancer deaths. The M/I ratio was derived from the data of 32 regional population-based cancer registry sites between 2003 and 2004 in China. The M/I ratio was calculated using Poisson regression adjusted for age, gender and cancer registry sites [16].

### Exposure Data for Risk Factors of Esophageal Cancer in China

The current health effects of risk factors reflect past patterns of exposure to these risk factors [17]. An average latency time of 15 years for risk factors and esophageal cancer occurrence is a reasonable assumption. Therefore, priority was given to exposure data in 1990, or close to that year.

#### Prevalence of tobacco smoking in China

Data on smoking prevalence were abstracted from the results of two National Smoking Surveys in China (in 1984 and 1996). Briefly, 29 provinces, autonomous regions, and municipalities directly under the Central Government were included in the 1984 national survey [18]. About 519,600 subjects (including 258,422 men and 261,178 women) were investigated using stratified random sampling. The overall prevalence of smoking was 33.9%, with 61.0% in men and 7.0% in women. In 1996, Gonghuan Yang et al [19] conducted a population-based survey of smoking involved 145 disease surveillance points in 30 provinces of China to survey 120,298 persons (including 63,793 men and 56,020 women) using a 3-stage cluster, random sampling method. The overall smoking prevalence was 37.6%, with 66.9% in men and 4.2% in women. Smoking prevalence in 1990 was estimated by linear interpolation using the results of these two national surveys.

#### Prevalence of alcohol drinking in China

Data on the prevalence of alcohol consumption were obtained from the 1991 National Hypertension Survey of China [20], which covered 30 provinces, autonomous regions, and municipalities of China. Multistage cluster random sampling was used to survey 949,539 subjects over 15 years old.

#### Dietary factors

Data on exposure prevalence were derived from the Chinese health and nutrition survey (CHNS) in 1991, covering 8 representative provinces (48 cities and counties) that varied substantially in geography, economic development, public resources, and health indicators and was carried out by the Chinese Academy of Preventive Medicine and the University of North Carolina Population Center [21]. In this 1991 nutrition survey, fruit and vegetable intakes were treated as continuous variables, and were defined as the mean per capita dietary intake of fruit and vegetables, measured in grams per day (g/d). In our study, we categorized the consumption of fruit and vegetables in quintiles, separately by regions (urban and rural) and genders.

Some risk factors of esophageal cancer (e.g. poverty, history of head and neck cancer, frequent consumption of hot beverages, etc.) were not included in our estimates, because few data were available in the Chinese population at large.

### Relative Risk (RR)

To evaluate the RR of risk factors and esophageal cancer in the Chinese population, we conducted a systematic search of publications in: PubMed, Websites, VIP Information, China National Knowledge Infrastructure (CNKI) and other databases (including Elsevier, Science Direct, and Springer). The search words included “smoking”, “tobacco smoking”, “alcohol drinking”, “alcohol consumption”, “vegetables”, “low vegetable intake”, “low vegetable consumption”, “fruit”, “low fruit intake”, “low fruit consumption” “meta-analysis”, “case-control study”, “cohort study” and “esophageal cancer”. Inclusion criteria for studies were: a. The studies should contain relative risks or odds ratios and corresponding 95% confidence intervals; b. The highest priority was given to meta-analyses and large-scale surveys of representative samples of China. If such studies were not available, non-representative samples of Chinese population studies were used. If Chinese data were not available, the final option was to use meta-analyses from other Asian or non-Asian countries.

We abstracted RRs separately for men and women; however, RRs were available for women in China in only a few studies, resulting in statistical instability of risk estimates. Therefore, if the RRs for women were not available (such as the RR for smoking or alcohol drinking), we used the corresponding RRs for men to estimate for both genders.

RR for smoking was obtained from a cohort study in China [22] and RR for alcohol drinking was abstracted from a large Chinese meta-analysis [23].

RRs for vegetable and fruit intake were calculated from Asian studies, including a large meta-analysis on the dose-response relationship of fruit and vegetable intake and cancer incidence [24], which provided RRs for an additional intake of 100 g/d. The summary RRs from these Asian studies were 0.68 (95% CI: 0.43, 1.06) for fruit intake and 0.98 (95% CI: 0.91, 1.05) for vegetable consumption. In our estimates, these RRs were first transformed into a log scale and divided by 100 to get the log RR/gram per day, and then multiplied by the lower limit of every quintile of fruit or vegetable consumption. Finally, we divided the RRs in the other quintiles by that in quintile 5 (Q5) to obtain final estimates, and we assumed that the RR in Q5 was equal to 1.

### Statistical Analysis

PAF is defined as a proportion of cancers in the total population that is attributable to a risk factor. PAF can be calculated by the following formula, as that by Levin [25]:


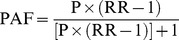


RR is relative risk of a risk factor (such as smoking or alcohol drinking) and esophageal cancer, while P is the prevalence of exposure to the risk factor in the total population.

For low vegetable and fruit intake, PAF was estimated for a shift of all to the top quintile that is a full shift, by the following formula.


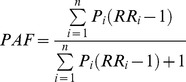


RR_i_ is the relative risk of quintile i (i = 1, 2, 3, 4, 5). *P_i_* is the prevalence of quintile i in the full shift. The full shift includes a shift of 4 quintiles for the top 20%, 3 quintiles for the next 20% etc.

The combined PAF for exposure to smoking, alcohol drinking and low vegetable and fruit intake can be calculated by the following formula [26]:


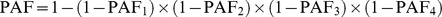


where PAF_1_ is the PAF for exposure to smoking, PAF_2_ is the PAF for exposure to alcohol drinking, PAF_3_ is the PAF for exposure to low vegetable intake, and PAF_4_ is the PAF for exposure to low fruit intake. This formula assumes independence of exposure from the four sources.

## Results

Esophageal cancer killed 190,233 people (131,685 men and 58,548 women) in China in 2005. The number of incident esophageal cancer cases during this year was calculated to be 236,589, including 163,361 in men and 73,228 in women.

### Estimates of Prevalence

The estimated overall prevalence of smoking in 1990 was 35.8%, with 64.0% in men and 5.6% in women, respectively. The overall prevalence of alcohol drinking was 17.9%, with 35.1% in men and 2.6% in women, respectively. In urban areas of China, the highest quintile level of vegetable intake was ≥441.7 g/d among men and ≥416.7 g/d among women, respectively, while the highest quintile of fruit intake was 150.0 g/d in both genders. In rural areas of China, both men and women consumed slightly more vegetables and slightly less fruit than their urban counterparts.

### Estimates of Relative Risk

The RR for smoking (for both men and women) was 1.34 (95% CI: 1.16–1.54). For alcohol drinking, the RR (for both men and women) was 1.51 (95% CI: 1.18–1.30). For the lowest quintile of vegetable intake, the RR was very similar (1.09–1.10) among both urban and rural men and women. For the lowest quintile of fruit intake, the RR was 1.78 among urban men and women, it was 1.67 among rural men, and it was 1.72 among rural women (Table 1).

**Table 1:** Relative risks (RR) between selected risk factors and esophageal cancer, used in the calculation of Population Attributable Fractions.

Risk factors	RR			Source
	Men	Women	Design	
Smoking	1.34	1.34	Cohort study	[18], [19]
Alcohol drinking	1.51	1.51	Meta-analysis	[20]
Vegetable intake*: urban			–	[15]
Quintile 5	1.00	1.00		
Quintile 4	1.02	1.02		
Quintile 3	1.04	1.04		
Quintile 2	1.06	1.05		
Quintile 1	1.09	1.09		
Vegetable intake*: rural			–	[15]
Quintile 5	1.00	1.00		
Quintile 4	1.03	1.02		
Quintile 3	1.04	1.04		
Quintile 2	1.06	1.06		
Quintile 1	1.10	1.09		
Fruit intake*: urban			–	[15]
Quintile 5	1.00	1.00		
Quintile 4	1.21	1.21		
Quintile 3	1.38	1.42		
Quintile 2	1.57	1.57		
Quintile 1	1.78	1.78		
Fruit intake*: rural			–	[15]
Quintile 5	1.00	1.00		
Quintile 4	1.29	1.27		
Quintile 3	1.38	1.42		
Quintile 2	1.47	1.51		
Quintile 1	1.67	1.72		

* Our estimates of RRs were obtained from a meta-analysis and were first transformed into a log scale and divided by 100 to get the log RR/gram per day, and then multiplied by the lower limit of every quintile of vegetable or fruit consumption. Finally, we divided the RRs in the other quintiles by that in quintile 5 (Q5) to obtain final estimates, and we assumed that the RR in Q5 was equal to 1. The details of this RR calculation for low vegetable and fruit intake have been described elsewhere [15].

### PAF Calculations

Tobacco smoking was responsible for 23,572 deaths from esophageal cancer among men (PAF = 17.9%) and 1,112 deaths among women (PAF = 1.9%) (Table 2). Corresponding figures for esophageal cancer incidence were 29,242 among men and 1,391 among women. The fraction of esophageal cancer caused by alcohol drinking was 15.2% for men and 1.3% for women. Our estimates showed that 4.3% of esophageal cancer deaths and cases among men (PAF = 4.1% for urban men and 4.4% for rural men) and 4.1% among women (PAF = 3.9% for urban women and 4.1% for rural women) were attributable to low vegetable intake, respectively. Low fruit intake was responsible for 27.1% of esophageal cancer deaths and cases among men (PAF = 28.0% for urban men and 26.6% for rural men) and 28.0% among women (PAF = 28.5% for urban women and 27.8% for rural women). We estimate that 87,065 (45.8%) of the total esophageal cancer deaths, including 67,686 (51.4%) of the deaths in men and 19,379 (33.1%) of the deaths in women, and 108,206 incident esophageal cancer cases (83,968 in men and 24,238 in women) in 2005 were attributable to the combined effects of smoking, drinking, low vegetable intake and low fruit intake.

**Table 2:** Esophageal cancer deaths and cases attributable to smoking, drinking, low vegetable intake and low fruit intake in China in 2005.

Risk factors	Men			Women			Total		
	PAF†(%)	Deaths	Cases	PAF†(%)	Deaths	Cases	PAF†(%)	Deaths	Cases
Smoking	17.9	23,572	29,242	1.9	1,112	1,391	13.0	24,684	30,633
Drinking	15.2	20,016	24,831	1.3	761	952	10.9	20,777	25,783
Low vegetable intake	4.3	5,662	7,025	4.1	2,400	3,002	4.2	8,062	10,027
Low fruit intake	27.1	35,686	44,271	28.0	16,393	20,504	27.4	52,079	64,775
Total*	51.4	67,686	83,968	33.1	19,379	24,238	45.8	87,065	108,206

* Combined PAF for smoking, drinking, low vegetable intake and low fruit intake and esophageal cancer was calculated using the following formula: PAF = 1−(1−PAF_1_)×(1−PAF_2_)×(1−PAF_3_)×(1−PAF_4_).

† PAF = Population Attributable Fraction.

## Discussion

This study provides an estimate of the contribution of four known environmental risk factors to the occurrence of esophageal cancer in China in the year 2005. We estimate that 45.8% of esophageal cancer deaths were attributable to the combined effects of tobacco smoking, alcohol drinking, low vegetable intake and low fruit intake. The deaths in China attributable to these causes in 2005 represent about 21% of all esophageal cancer deaths worldwide in that year [1]. For smoking and drinking, the PAFs for men were much higher than those for women, while for low vegetable intake and low fruit intake, PAFs were similar in both genders.

In our study, 17.9% of esophageal cancer deaths among men and 1.9% among women were attributable to tobacco smoking, respectively. These estimates were comparable to the few previous estimates from Chinese studies. A large-scale retrospective proportional mortality study by Liu and colleagues [27] estimated that tobacco smoking was responsible for 27.9% of esophageal cancer deaths in middle-aged men and 2.8% in middle-aged women. A more recent prospective cohort study, conducted by Gu and colleagues [28], showed that the fraction of esophageal cancer deaths caused by tobacco smoking was 19.4% in men and 1.6% in women. Our estimates are lower than those of Liu’s study [27], but similar to those of Gu’s study [28]. The reasons for the differences in these estimates are probably related to differences in the RRs and smoking prevalences used in calculating them (Table 3). The RR used in our study was obtained from a prospective study with 29,584 adults participating in general trial cohort in China, and was the same RR as that found in men in Gu’s study (RR = 1.34). The RR in Liu’s study was higher in men (1.61), possibly because the smoking information was derived retrospectively from the surviving spouse or family members, which may have led to misclassification or recall biases. In our study, smoking prevalence was estimated by linear interpolation using the results of the 1984 and 1996 national smoking surveys, while in Gu’s study it was abstracted from a cross-sectional study conducted in 2000–2001 [29]. In Liu’s study, information of smoking habits was derived from the family members.

**Table 3:** Comparison of relative risk, prevalence and population attributable fraction (PAF) for smoking and esophageal cancer in three studies from China.

Studies	Relative risk		Prevalence (%)		PAF (%)	
	Men	Women	Men	Women	Men	Women
Our study	1.34	1.34	64.0	5.6	17.9	1.9
Liu’s study [27]	1.61	1.34	63.4*	8.5*	27.9	2.8
Gu’s study [28]	1.34	1.24	60.2†	6.9†	19.4	1.6‡

* Smoking prevalence was calculated from relative risk and PAF.

† Smoking prevalence came from the international collaborative study of cardiovascular disease in Asia [29].

‡ PAF was estimated using the following formula: 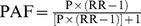.

Alcohol drinking is another important risk factor for esophageal cancer. We estimated that the fraction of esophageal cancer in our population that was attributable to alcohol drinking was 15.2% for men and 1.3% for women. Danaei and his colleagues [30] calculated mortality from 12 types of cancer attributable to nine risk factors in seven World Bank regions in 2001, and showed that 24% of all esophageal cancer deaths (men and women combined) in low and middle-income countries and 41% in high income countries were attributable to alcohol drinking. Our estimate among men was lower than the estimate of this global analysis for both men and women in low and middle income countries, and was much lower than that in high income countries. For women, our PAF was much lower than the estimate from a previous population based case-control study in the US (64.4%) [31] and the estimate from a previous European population based prospective cohort study (25% for cancers of the upper aerodigestive tract) [32]. In China, however, an increase in the prevalence of alcohol drinking among women between 1991(2.6%) and 2002 (4.5%) was observed. Thus it is expected that the contribution of alcohol drinking to the cancer burden will increase in the future.

Most previous studies about the cancer burden in global or national studies have evaluated the combined effect of total fruit and vegetable intake [30], [31], [33]. Our report separately estimates the esophageal cancer burden attributable to low intake of fruit and low intake of vegetables. Fruit and vegetables contain different nutrients and have different nutritional values [34]. For example, the contents of vitamins, minerals, fiber, and phytochemicals are higher in most vegetables, especially in dark vegetables, while carbohydrates and organic acids are higher in fruits. Danaei and his colleagues [30] calculated that 19% of esophageal cancer deaths in low and middle-income countries were attributable to low vegetable and fruit intake. Our estimates indicated a little higher proportion of esophageal cancer deaths were attributable to low vegetable and fruit intake, reflecting the different sources of RRs and exposure rates in the two studies.

We compared our estimates of the combined effects of smoking, drinking, and low fruit and vegetable intake with similar estimates published from previous studies in different countries (Table 4). PAFs in our analysis were much lower than the corresponding figures in the USA [31], France [8], the Nordic countries [9] and the UK [10]. Tobacco smoking and alcohol drinking are the major risk factors for esophageal cancer in these western populations, accounting for around 80% of the population attributable risk. In China, on the other hand, smoking and drinking are less important risk factors for esophageal cancer.

**Table 4:** Comparison of the population attributable fraction (PAF, %) of esophageal cancer deaths or new cases attributable to the combined effects of smoking, drinking, low vegetable and fruit intake, overweight/obesity, ionizing radiation and occupation in various studies.

Studies	Men	Women	Total
Our study*	51.4	33.1	45.8
Worldwide study [30] *	–	–	62.0
Low and middle incomecountries	–	–	58.0
High income countries	–	–	85.0
US study [31] *	92.8	87.7	89.4
French study [8] †	79.2	49.4	74.2
Nordic study [9] ‡	96.0	74.0	89.0
UK study [10] •	89.7	88.2	89.0

* PAF of esophageal cancer was calculated for the combined effects of smoking, drinking and low vegetable and fruit intake.

† PAF of esophageal cancer was estimated for the combined effects of smoking, drinking and overweight/obesity.

‡ PAF of esophageal cancer was estimated for the combined effects of smoking and drinking.

• PAF of esophageal cancer was calculated for the combined effects of smoking, drinking, low vegetable and fruit intake, overweight/obesity, ionizing radiation and occupation.

This study had several limitations. First, we did not evaluate the effects of some other known risk factors, including overweight/obesity, tooth loss and poor oral health, and consumption of hot drinks. BMI has been inversely associated with esophageal squamous cell carcinoma in various countries, including China [35]. At least two prospective studies in China have also shown that tooth loss increases risk of developing upper gastrointestinal cancers [36], [37]. And in the Golestan Case-Control Study in Iran, drinking hot and very hot tea were associated with 2-fold and 8-fold elevated risks of esophageal cancer, respectively [38]. Therefore, there is a need for additional studies to evaluate the contribution of these and other risk factors to the burden of esophageal cancer in China. Second, there were some sources of uncertainty in the relative risks we used in making our PAF estimates. The RRs of alcohol drinking and of low vegetable and fruit intake were obtained from meta-analyses, and some of the original studies included in these meta-analyses may not have been adjusted for confounding factors. Moreover, the RRs of low vegetable and fruit intake were derived from non-Chinese Asian studies, and there was uncertainty in the extrapolation of these RR to the Chinese population. It was difficult to evaluate the direction and potential magnitude of these potential biases. Also, RRs in men were used for both genders because of a lack of available data among women or the statistical instability of risk estimates in women, but RRs for men and women might be different. Third, we were not able to collect information on the amount and duration of smoking or alcohol drinking in the Chinese population at large, and epidemiological studies have demonstrated that these variables are important to fully assess the impact of these exposures on esophageal cancer [39], [40]. Fourth, we did not adjust our estimates of PAF for the possible interaction because too little data are available in studies from China to provide valid estimates of the ORs of interaction between different risk factors. Finally, another potential limitation was the lack of measured esophageal cancer incidence data in China, which meant that we had to estimate these numbers from mortality data and a mortality-to-incidence ratio. This M/I ratio was calculated by Poisson regression models, adjusted for age, gender and cancer registry site, but it was not adjusted for other relevant factors such as socioeconomic status because of a lack of available data.

In summary, our results provide an assessment of the burden of tobacco smoking, alcohol drinking, low vegetable intake and low fruit intake on the occurrence of esophageal cancer in China. About 46% of esophageal cancer deaths in 2005 were caused by these four modifiable environmental risk factors. A new strategic program called “Healthy China 2020” has been developed by the Minister of Health in China [41], targeting key health problems through a series of national interventions and practical public health actions. The present estimates provide basic data that are important for guiding such policy-makers on issues of esophageal cancer prevention and control.
